# Response of bacterial community composition and co-occurrence network to straw and straw biochar incorporation

**DOI:** 10.3389/fmicb.2022.999399

**Published:** 2022-09-30

**Authors:** Mingcheng Du, Jianyun Zhang, Guoqing Wang, Cuishan Liu, Zhenlong Wang

**Affiliations:** ^1^State Key Laboratory of Hydraulic Engineering Simulation and Safety, Tianjin University, Tianjin, China; ^2^State Key Laboratory of Hydrology-Water Resources and Hydraulic Engineering, Nanjing Hydraulic Research Institute, Nanjing, China; ^3^Yangtze Institute for Conservation and Development, Nanjing, China; ^4^Research Center for Climate Change, Nanjing, China; ^5^Wudaogou Experimental Station for Hydrology and Water Resources, Bengbu, China; ^6^Anhui Hydraulic Research Institute, Huai River Commission, Bengbu, China

**Keywords:** straw and straw biochar, soil bacterial community, co-occurrence network, keystone taxa, lime concretion black soil, maize

## Abstract

Microbial decomposition plays a crucial role in the incorporation of straw and straw biochar (SSB) into soil. Lime concretion black soil (LCBS) is a typical low-medium crop yield soil, and it is also one of the main soil types for grain production in China. However, the link between SSB additions and soil bacterial communities in LCBS remains unclear. This study explored the effects of SSB incorporation on bacterial community composition, structure and co-occurrence network patterns at different soil depths and maize growth stages. The results showed that soil PH, soil organic matter and total nitrogen significantly affected the seasonality and stratification of the soil bacterial community. The composition and diversity of bacterial communities were significantly affected by growth period and treatment rather than soil depth. Specifically, the bacterial community diversity increased significantly with crop growth at 0–20 cm, decreased the relative abundance of Actinobacteria, and increased the relative abundance of Proteobacteria and Acidobacteria. SF (straw with fertilizer) and BF (straw biochar with fertilizer) treatments decreased bacterial community diversity. Co-occurrence networks are more complex in BF, S (straw), and SF treatments, and the number of edge network patterns is increased by 92.5, 40, and 60% at the maturity stage compared with F (fertilizer) treatment, respectively. Moreover, the positive effect of straw biochar on the bacterial network pattern increased with time, while the effect of straw weakened. Notably, we found that rare species inside keystone taxa (Gemmatimonadetes and Nitrospirae) play an indispensable role in maintaining bacterial network construction in LCBS. This study offers a comprehensive understanding of the response of soil bacterial communities to SSB addition in LCBS areas, and provides a reference for further improvement of LCBS productivity.

## Introduction

The application of chemical fertilizers in agricultural production has become very common and essential (Zhang et al., 2019). Different types of chemical fertilizers have been applied to farmland over the past few decades, and the long-term application of chemical fertilizers can have negative effects on soil, such as soil degradation and lower productivity (Adesemoye and Kloepper, 2009; Agegnehu et al., 2017). As a renewable resource, straw is rich in nutrients, and its return to the field is considered to be an effective and promising method to improve soil quality and increase soil fertility (Dikgwatlhe et al., 2014; Ma et al., 2019). Its addition not only changes the soil’s physicochemical properties, but also affects the living environment of the microbial community in the soil (Navarro-Noya et al., 2013).

Microorganisms are an important part of the soil ecosystem and are the main participants in soil physicochemical processes. They include highly diverse and complex bacteria, fungi, viruses and protozoa, of which bacteria are the most abundant (Takaku et al., 2006; Bardgett and van der Putten, 2014; de Araujo et al., 2018). Straw returning to the field provides a carbon source that is easily decomposed for microorganisms, promoting the reproduction and enriching the species of those microorganisms (Morais et al., 2019). Previous studies have shown that bacteria (such as Proteobacteria, Actinobacteria, and Acidobacteria) dominate the microbial community during the early stages of straw decomposition (Liu et al., 2019). Straw returning significantly increases bacterial biomass compared with no returning (Navarro-Noya et al., 2013; Chen et al., 2017). Morais et al. (2019) concluded that straw returning had a significant effect on soil bacteria. However, it has also been suggested that the addition of straw with a high C/N ratio leads to a shortage of nitrogen, which intensifies the fierce competition for nitrogen between the microbial community and plants, thereby inhibiting the growth of microorganisms (Kuzyakov and Xu, 2013).

As research on the effect of straw returning on soil quality has deepened, researchers have found that the decomposition rate of straw is slow (Brunetto et al., 2011), which increases farmland diseases and insect pests (Liu et al., 2022a). Therefore, some scholars have proposed generating straw biochar through high-temperature pyrolysis and then returning it to the field (Laird, 2008; Feng et al., 2021). The incorporation of biochar changes the soil’s physicochemical properties and has a direct or indirect impact on soil microbial activities (Wu et al., 2019). This positive effect is related to the porous structure and large specific surface area of biochar, which provides a suitable and favorable shelter for the microbial community while also providing unstable carbon-containing organic matter and nutrients for microbial growth and reproduction, changing microbial activity (Gomez et al., 2014; Jaafar et al., 2014; Partovi et al., 2021). Previous reports have suggested that short-term biochar addition can significantly alter soil bacterial diversity, and a combination of biochar and chemical fertilizers is considered the most beneficial treatment for soil bacteria (Yu et al., 2018b). But it does not apply to all soils and the effect is not always positive (Zhang et al., 2019). The effect of biochar on microbes is not certain and the diversity of biochar and soil types means the practical effect in different soil types cannot be generalized (Jeffery et al., 2015; Pan et al., 2016; Ginebra et al., 2022). This positive or negative effect is not clear but we do think it might be related to changes in chemical properties after biochar pyrolysis, soil PH and nutrients (Paz-Ferreiro et al., 2011; Gomez et al., 2014). In addition, most of the effects of biochar on soil microorganisms were obtained based on specific sampling times, and these results focused on specific states of soil bacteria, with few effects reported for different growth stages of crops (Zhen et al., 2014; Bello et al., 2021). Consequently, time course investigations are necessary to indicate changes in soil bacterial communities due to straw or straw char addition (Bello et al., 2021).

The North Anhui Plain is one of the important grain producing areas in China. The main soil type is lime concretion black soil (LCBS), which accounts for 54% of the total area of the region (Du et al., 2021). It is recognized as a medium and low-yielding soil, largely because of its unique dry shrinkage and wet expansion characteristics and poor water and fertilizer retention properties. Previous studies have shown that straw returning can improve the LCBS properties, such as soil bulk density and soil water content (Ruan et al., 2022). However, little is known about the effects of straw and equal proportions of straw biochar incorporation on soil microbial communities. In addition, the effects of the incorporation of straw and straw biochar (SSB) on the co-occurrence network and keystone taxa (KT) changes of soil bacteria in different growth stages of maize are still unclear. The main purpose of this study is threefold: (1) evaluate the effects of different treatments and soil depths on soil bacterial diversity and richness in growth stages of maize, (2) determine whether soil bacterial network patterns, topological properties, and KT are affected by different treatments in growth stages of maize, (3) investigate the links between environmental variables and soil bacterial genera and KT to discover the key factors mediating bacterial species succession.

## Materials and methods

### Study site description

The field experiment was conducted at the Wudaogou Hydrological Experiment Station in Guzhen County, Bengbu City, Anhui Province (117°21′ E, 33°9′ N), which is located in the southern part of the North Anhui Plain and is an important seed crop base for maize and wheat in China. The area is a transition zone between warm temperate and north subtropical climates. The annual average water surface evaporation, temperature and rainfall is 1,011 mm, 14.9 °C, and 885 mm, respectively, and the rainfall from June to September accounts for 60–70% of the total annual rainfall. The soil type is LCBS. The basic soil properties at 0–20 cm depth at the beginning of the experiment is shown in Table 1.

**TABLE 1:** Basic properties of soil at 0–20 cm.

Soil depth (cm)	PH	Total nitrogen (mg⋅kg^–1^)	Total phosphorus (mg⋅kg^–1^)	Available phosphorus (mg⋅kg^–1^)	Organic matter (g⋅kg^–1^)	Bulk density (g⋅cm^–3^)	Soil particle distribution (%)		
							<0.002 mm	0.002–0.02 mm	0.02–2 mm
0–10	7.5	876.0	561.9	9.9	15.1	1.18	15.1	23.7	61.3
10–20	7.6	867.0	537.2	6.1	13.4	1.60	14.7	22.4	62.9

### Field experiment

In the experiment, four field treatments were set up in randomized blocks: straw biochar with fertilizer (BF), straw (S), fertilizer (F), and straw with fertilizer (SF). Each plot was divided into three subplots with three replicates (133.3 m^2^). Soil pits were dug around the plots and an aluminum-plastic board was inserted vertically with 20 cm of the board above the ground to avoid the influence of soil moisture and nutrients from other plots in the field. The stubble and straw were removed manually from the plot when the wheat was harvested. Wheat straw was crushed into 3–5 cm and returned to the field using a pulverizer, and the application amount was 5,400 kg⋅ha^–1^. The raw material of the biochar was wheat straw, and the application amount was 1,800 kg⋅ha^–1^ according to the straw conversion ratio of 33%. The wheat straw was purchased from Zhengzhou Jinbang Environmental Technology Co., Ltd. On June 12, 2021, SSB was evenly spread in the soil surface of the test plot, and the rotary tiller was evenly rotated into the soil tillage layer. Base fertilizer urea (N 46.4%) and compound fertilizer (N-P_2_O_5_-K_2_O = 30-5-5%) were applied separately, and the application amounts were 15 and 60 g⋅m^–2^, respectively. On the same day, maize was artificially sown with 60 cm between each row and 27 cm between each plant. The tested maize variety was Denghai 533. When maize was sown, an open space (buffer zone) of 60 cm from the edge of the plot was left to avoid the influence of adjacent plots. All experimental plots had the same, conventional field management, such as weeding and insecticide application.

### Sampling and soil physicochemical determination

Soil samples were collected at the seedling stage (SS) and maturity stage (MS) of maize using a soil auger (diameter = 5 cm), and each subplot at a depth of 0–20 cm (0–10 cm and 10–20 cm) was randomly sampled according to the five-point method. Soil samples were collected between two maize plants. Five soil samples were collected from each layer, and after manual removal of visible stones or plant debris, they were thoroughly mixed to make one composite sample. To reduce the influence of the external environment on microorganisms, sample collection was divided into two sessions. About 10 g of soil samples were collected and placed into sealed bags the first time. The samples were then placed in a foam box with dry ice, and transported to the laboratory and stored at −80°C for microbial DNA extraction. About 1,000 g of soil samples were taken during the second session to determine the soil’s physicochemical properties, including ammonia nitrogen (NH_4_^+^-N), nitrate nitrogen (NO_3_^–^-N), total nitrogen (TN), available phosphorus (AP), total phosphorus (TP), organic matter (SOM), bulk density (BD), PH, and soil water content (SWC).

TN was determined by the Kjeldahl method. Soil NH_4_^+^-N and NO_3_^–^-N were extracted in a KCl solution and then determined using spectrophotometry (Lu, 2000). The soil AP was determined by using the molybdenum-antimony anti-colorimetric method after extraction with N_*a*_HCO_3_ solution. TP was determined by alkali fusion-molybdenum antimony anti-spectrophotometry. The SOM was converted by measuring the organic carbon content with TOC analyzer (XPERT-TOC/TNb, Trace Elementa, Netherlands). Soil PH was measured using a PH meter (DZS-706, INESA, Shanghai, China) in a solution with a soil/water ratio of 1:2.5 (*w*/*v*). Soil bulk density (BD) was obtained by the cutting ring method (Lu, 2000). The SWC was determined by the drying method (105°C).

### DNA extraction and high-throughput sequencing

DNA samples were extracted using the DNeasy PowerSoil Kit (QIAGEN, Inc., Netherlands) in line with the manufacturer’s instructions. DNA concentration, quantity and quality were determined using a NanoDrop ND-1000 spectrophotometer (Thermo Fisher Scientific, Waltham, MA, USA) and 1.2% agarose gel electrophoresis. We performed PCR amplification of the V3-V4 region of the bacterial 16S rRNA gene by using the forward primer (338F: 5′-ACTCCTACGGGAGGCAGCA-3′) and the reverse primer (806R: 5′-GGACTACHVGGGTWTCTAAT-3′) (Ding et al., 2021). The amplification conditions were as follows: 98°C for 2 min, followed by 25 cycles consisting of 98°C for 15 s, 55°C for 30 s, and 72°C for 30 s, and a final extension at 72°C for 5 min. The final product was purified and quantified using Agencourt AMPure Beads (Beckman Coulter, Indianapolis, IN) and PicoGreen dsDNA Assay Kit (Invitrogen, Carlsbad, CA, USA), respectively. And equimolar pooling and paired-end sequencing (2 × 300 bp) were performed on the Illumina MiSeq platform (Ding et al., 2021). Furthermore, all raw reads were analyzed on the QIIME2 platform (Knight et al., 2010). The raw data were quality controlled, denoised, merged, and chimeras removed before DADA2 clustering (Callahan et al., 2016). Amplicon sequence variants (ASVs) were labeled by the ASV number in this study.

### Statistical analyses

The alpha diversity of microbial communities (Chao1 and Shannon indices) was estimated using QIIME2. The significant effects of growth stage, treatment, soil depth and their interactions on microbial alpha diversity were determined using three-way analysis of variances (ANOVAs). The least significant difference (LSD) *post hoc* test identified significant differences between treatments in microbial alpha diversity. Comparison of bacterial beta diversity by Bray-Curtis distance was based on non-metric multidimensional scaling (NMDS) analysis and analysis of similarities (ANOSIM) using the “vegan” package (v.2.5-7) in R (v.4.0.2). The student’s t-test was used to evaluate differences between bacterial taxonomic levels under specific treatments. Correlations between soil properties and bacterial genera were analyzed by redundancy analysis (RDA), which was performed in the vegan package. Multicollinearity tests were used to remove variables with variance inflation factors (VIF) >10 (Aertsen et al., 2012), and finally, permutation tests (999 permutations) were performed to assess the significance of soil properties on bacterial communities. Network analysis was carried out using the “psych” package (v.2.1.9) and the “igraph” package (v.1.2.6) in R to explore bacterial network patterns (Csardi et al., 2006; Röttjers and Faust, 2018). The co-occurrence network was constructed based on the spearman correlation coefficient >0.7 and FDR-corrected *P*-values <0.05 (Ge et al., 2021; Liu et al., 2022b). The topological features of each network were calculated using the igraph package. The KT identified included high average degree, high betweenness centrality, and high closeness centrality (Banerjee et al., 2018; Ge et al., 2021). The spearman correlations between soil properties and bacterial KT were calculated and heatmaps were drawn using the psych package and the “corrplot” package (v.0.92) in R.

## Results

### Gene sequence data characteristics

A total of 2,058,101 bacterial non-singleton sequences were obtained from 48 soil samples, and the average read length of bacterial sequences was 417 bp (the main length ranged from 404 to 433 bp) after screening. We observed 39 phyla, 123 classes, 283 orders, 494 families, 1,027 genera, and 1,626 species in this study. The rarefaction analysis found that the number of recorded ASVs tended to be smooth, which indicated the amount of data in the sequence was reasonable (Supplementary Figure 1).

### Alpha and beta-diversity of bacterial communities under the incorporation of straw and straw biochar

**FIGURE 1:**
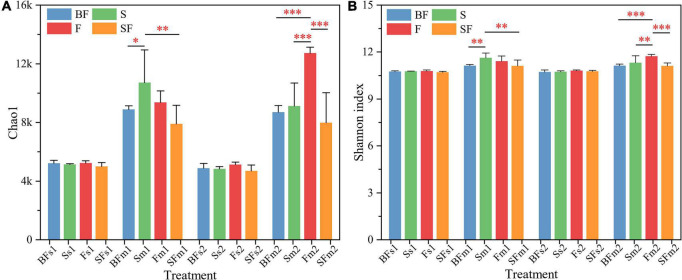
Changes in 0–20 cm soil bacterial alpha diversity (Chao1 and Shannon indices) during maize growth. **(A)** Chao1 and **(B)** Shannon index. BF, S, F, and SF stand for: straw biochar with fertilizer, straw, fertilizer, and straw with fertilizer, respectively. s: Seedling stage; m: Maturity stage. 1: 0–10 cm; 2:10–20 cm. *p < 0.05, ^**^p < 0.01, ^***^p < 0.001.

We used Chao1 and Shannon indices to characterize the α-diversity of soil bacterial communities (Figure 1). The analysis of the results revealed that all treatments had little effect on the α-diversity of soil bacterial at the SS of 0–20 cm. Bacterial communities tended to increase in the α-diversity over time, as measured by the Chao1 and Shannon indices. In the 0–10 cm soil layer at MS, BF and SF treatments significantly decreased the α-diversity of soil bacteria, S treatment significantly increased the α-diversity of soil bacteria, and F treatment had no significant effect on the α-diversity of soil bacteria. The F treatment significantly increased the α-diversity of soil bacteria at 10–20 cm depth at MS. Table 2 shows soil bacterial α-diversity was significantly affected by maize growth stage (*p* < 0.001), followed by treatment (*p* < 0.01), but neither was affected by soil depth or growth stage and soil depth interaction. The interaction between growth stage and treatment, treatment and soil depth, and growth stage, treatment and soil depth had a significantly greater impact on Chao1 than on Shannon index.

**TABLE 2:** Summary of three-way ANOVAs results for bacterial alpha diversity.

	Growth stage		Treatment		Soil depth		G × T		G × D		T × D		G × T × D	
	*F*	*P*	*F*	*P*	*F*	*P*	*F*	*P*	*F*	*P*	*F*	*P*	*F*	*P*
Chao1	254.9	0.000	7.1	0.001	0.1	0.792	5.0	0.006	1.5	0.223	4.0	0.020	3.3	0.034
Shannon	95.2	0.000	5.2	0.005	0.0	0.970	3.4	0.028	0.0	0.968	1.6	0.215	1.1	0.352

**FIGURE 2:**
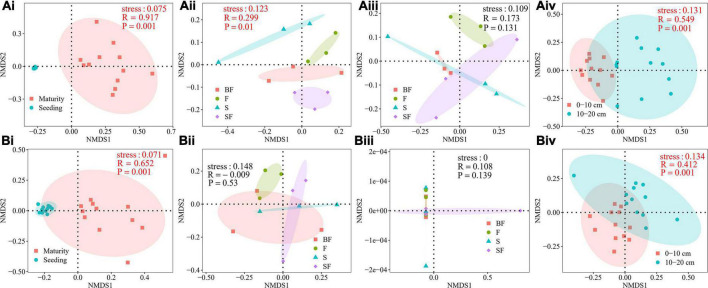
**(A)** Non-metric multidimensional scaling (NMDS) analysis of soil bacterial community composition at 0–10 cm depth. **(Ai)** growth stages, **(Aii)** different treatments at seedling stage, **(Aiii)** different treatments at maturity stage, **(Aiv)** different soil depths at seedling stage. **(B)** NMDS analysis of soil bacterial community composition at 10–20 cm depth. **(Bi)** growth stages, **(Bii)** different treatments at seedling stage, **(Biii)** different treatments at maturity stage, and **(Biv)** different soil depths at maturity stage.

NMDS analysis was performed to determine the effect of different treatments on the composition of the bacterial community at 0–20 cm depth in SS and MS (Figure 2). It revealed a clear difference in the soil bacterial community composition of maize at SS and MS (0–10 cm: *R* = 0.917, *p* = 0.001; 10–20 cm: *R* = 0.652, *p* = 0.001) (Figures 2Ai,Bi). At the same time, it revealed obvious differences in the bacterial community composition between 0–10 cm and 10–20 cm depths (SS: *R* = 0.549, *p* = 0.001; MS: *R* = 0.412, *p* = 0.001) (Figures 2Aiv,Biv). The bacterial community compositions of BF, S, F and SF treatments were significantly different only at the 0–10 cm depth at SS (Figures 2Aiii,Bii,iii). The results showed that sampling time and soil depth significantly affected soil bacterial community structure. Different treatments had little effect on soil bacterial community structure.

### Taxonomic classification and diversity of bacterial communities

**FIGURE 3:**
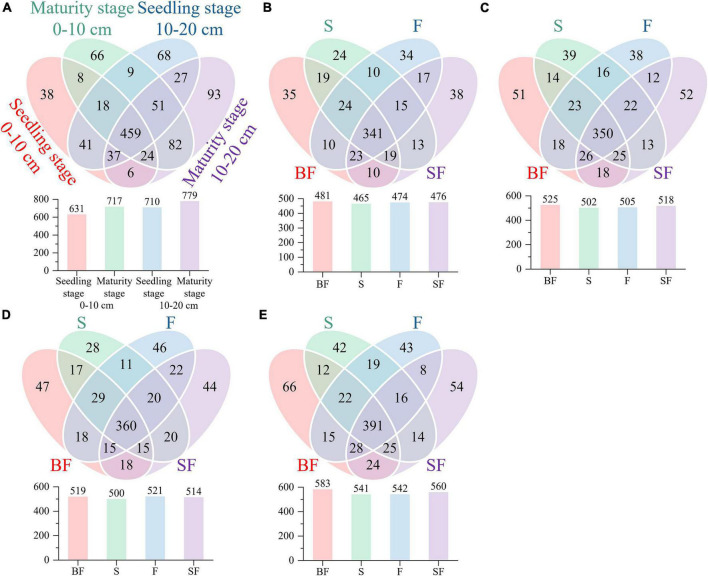
Comparing bacterial genera using Venn diagrams. **(A)** growth stages, **(B)** seedling stage, 0–10 cm, **(C)** maturity stage, 0–10 cm, **(D)** seedling stage, 10–20 cm, and **(E)** maturity stage, 10–20 cm.

**FIGURE 4:**
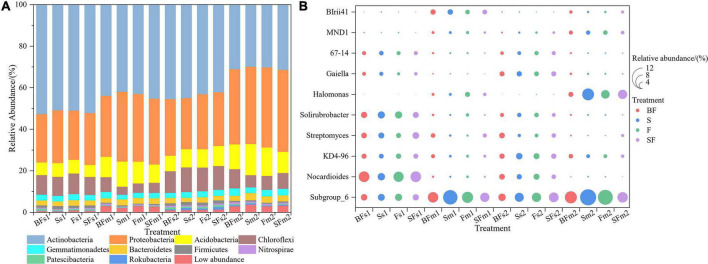
Relative abundance of taxonomic composition at the phylum **(A)** and genus **(B)** level of soil bacterial communities in 0–20 cm under straw and biochar addition. The top 10 relative abundances were shown. BF, S, F, and SF stand for: wheat straw biochar with fertilizer, wheat straw, fertilizer, and wheat straw with fertilizer, respectively. s: Seeding; m: Maturity. 1: 0–10 cm; 2: 10–20 cm.

We revealed some unique genera at different growth stages and different treatments. There were 786 and 880 genera in the samples collected at SS and MS, respectively, of which 159 (60%) unique genera were observed at the mature stage (Figure 3A). BF treatment produced the highest genera at SS and MS at the 0–10 cm and 10–20 cm depths, followed by SF treatment; BF and SF treatments produced more unique genera than F and S treatments (Figures 3B–E). The top 10 bacterial phyla and genera in relative abundance for all soil samples are shown in Figure 4. The soil bacterial community was dominated by Actinobacteria (42.36%), Proteobacteria (30.02%), Acidobacteria (9.49%) and Chloroflexi (8.26%) at the phylum level, accounting for about 90% of the relative abundance of all treatments. Compared with F treatment, the relative abundance of Actinobacteria in BF treatment and SF treatment slightly increased, and S treatment decreased. The relative abundances of Acidobacteria and Chloroflexi were reduced in BF and SF treatments. From SS to MS, the relative abundances of the dominant phyla Actinobacteria and Chloroflexi decreased, while the relative abundances of Proteobacteria and Acidobacteria increased. At SS, the dominant genera of soil bacteria at 0–20 cm depth included Subgroup_6, Nocardioides, KD4-96, Streptomyces and Solirubrobacter. However, only the relative abundance of Subgroup_6 increased significantly during the MS, and the relative abundance of several other dominant genera decreased significantly. There are also some genera with relative abundance below 1% that were not noticed at 10–20 cm, such as Nocardioides, Streptomyces, and Solirubrobacter.

**FIGURE 5:**
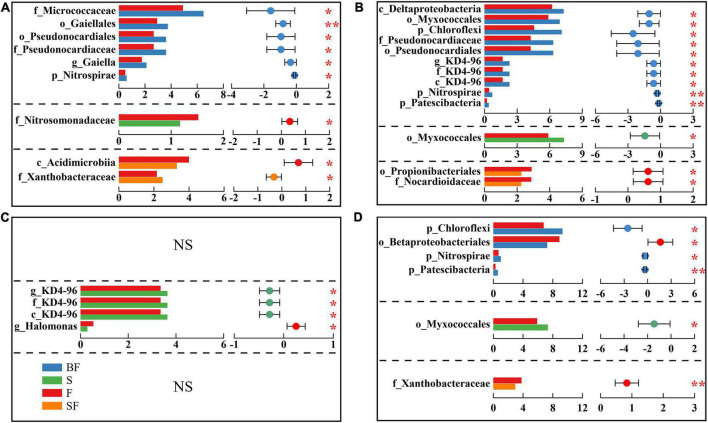
Inter-group difference test for relative abundance of bacteria. Panels **(A–D)** represent 0–10 cm seedling stage, 0–10 cm maturity stage, 10–20 cm seedling stage, and 10–20 cm maturity stage, respectively. The letters before bacterial names represent the abbreviations for different taxonomic levels (k-Kingdom, p-phylum, c-class, o-order, f-family, g-genus). The horizontal axis of histogram represents the average relative abundance of bacteria. Student’s *t*-test was used to assess significant differences between groups. The horizontal axis of the scatter plot represents the difference between the average relative abundances of bacteria for the two treatments, and the error bars are 95% confidence intervals. *p < 0.05, **p < 0.01.

Additionally, we identified differential bacteria between BF, S, SF treatments and F treatments using two-group comparisons and analyzed the mean relative abundance of differential bacteria (Figure 5). It can be found that the difference in soil bacterial community composition gradually increased with the growth of maize, with the difference in bacteria at different taxonomic levels greatest between BF and F treatments. For example, with the soil depth of 0–10 cm in SS (Figure 5A), BF treatment significantly increased o_Gaiellales by 28.2% (*p* < 0.01) and f_Micrococcaceae by 32.5% (*p* < 0.05); S treatment significantly decreased f_Nitrosomonadaceae by 23.3% (*p* < 0.05), and SF treatment significantly increased f_Xanthobacteraceae 15.5% (*p* < 0.05), which significantly reduced c_Acidimicrobiia by 17.4% (*p* < 0.05).

### Correlation between soil properties and bacterial community structure

**FIGURE 6:**
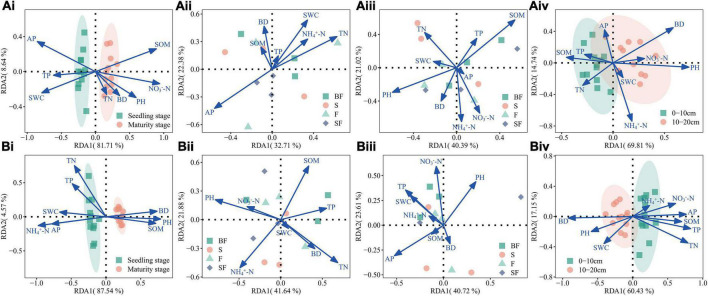
**(A)** Illustrating associations between bacterial genera and environmental variables at 0–10 cm depth based on redundancy analysis (RDA). **(Ai)** growth stages, **(Aii)** different treatments at seedling stage, **(Aiii)** different treatments at maturity stage, **(Aiv)** different soil depths at seedling stage. **(B)** Illustrating associations between bacterial genera and environmental variables at 10–20 cm depth based on RDA. **(Bi)** growth stages, **(Bii)** different treatments at seedling stage, **(Biii)** different treatments at maturity stage, and **(Biv)** different soil depths at maturity stage.

Based on RDA analysis, the results showed that soil physicochemical properties (NH_4_^+^-N, NO_3_^–^-N, TN, TP, AP, SOM, PH, BD, and SWC) affected bacterial communities at different growth stages, treatments and soil depths (Figure 6). The RDA1 and RDA2 had the highest overall interpretation rate for the observed changes in soil bacterial dynamics during the growth stages, reaching 90.4–92.1% (Figures 6Ai,Bi); the total interpretation rate for different soil depths is slightly lower, reaching 77.6–84.6% (Figures 6Aiv,Biv); the total interpretation rate for different treatments reached 55.1–64.3% (Figures 6Aii,iii,Bii,iii). It is worth noting that SOM (*r*^2^ = 0.576, *p*-value = 0.026) at 0–10 cm depth in MS, and TN (*r*^2^ = 0.544, *p*-value = 0.032) and PH (*r*^2^ = 0.533, *p*-value = 0.038) at 10–20 cm depth in SS were observed to be the main drivers that significantly affected and controlled bacterial communities (Supplementary Table 1).

### Co-occurrence network and keystone taxa assessments

**FIGURE 7:**
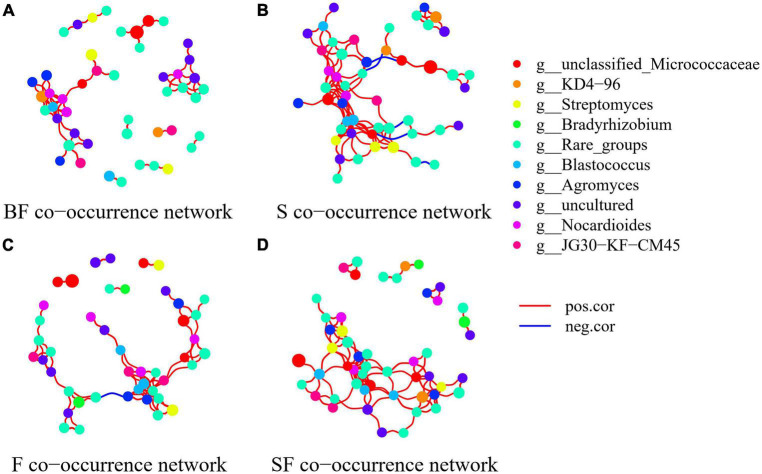
Network analysis showed the co-occurrence patterns of soil bacterial taxa at seedling stage in different treatments. **(A)** BF, **(B)** S, **(C)** F, and **(D)** SF. The size of each node is proportional to the relative abundance. The blue line represents a significant negative relationship (*p* < 0.05) between nodes, and the red line represents a significant positive relationship (*p* < 0.05) between nodes.

**FIGURE 8:**
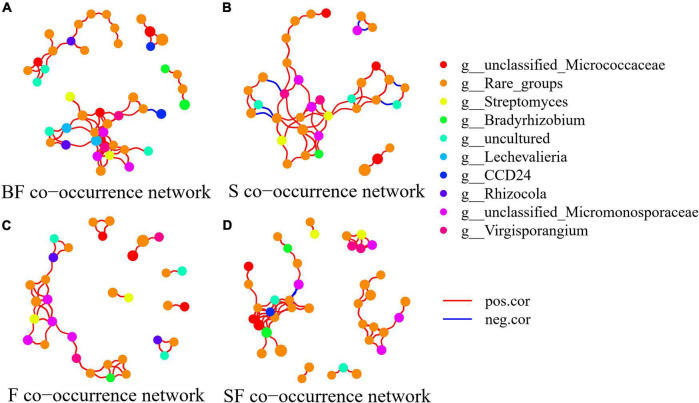
Network analysis showed the co-occurrence patterns of soil bacterial taxa at maturity stage in different treatments. **(A)** BF, **(B)** S, **(C)** F, and **(D)** SF. The size of each node is proportional to the relative abundance. The blue line represents a significant negative relationship (*p* < 0.05) between nodes, and the red line represents a significant positive relationship (*p* < 0.05) between nodes.

To determine the effect of straw and biochar incorporation on soil bacterial communities, we constructed a co-occurrence network of bacterial community composition under different treatments at SS and MS (Figures 7, 8). S and SF treatments increased bacterial community complexity at SS, but decreased bacterial community complexity in BF treatment (Figure 7). From SS to MS, the number of edges of network in S, F and SF treatments decreased by 46.7, 48.1 and 41.8%, respectively, reducing the complexity of bacterial community; but the number of edges of network increased by 24.2% in BF treatment (Figure 8; Table 3). The three treatments increased the number of edges in the bacterial network by 92.5% (BF), 40% (S), and 60% (SF) compared to the F treatment at MS, respectively (Table 3). SF treatment increased bacterial complexity compared to S treatment. Similarly, we found that whether SS or MS, the positive correlation between nodes and the number of edges showed a similar pattern. These results indicate that fertilizer (F) treatment and straw (S and SF) treatments reduced bacterial complexity as the crop grew, with the S and SF treatments being more affected than the F treatment. The decomposition of straw biochar increases the complexity of bacterial communities. BF, S, and SF treatments increased bacterial community complexity and positive associations in bacterial networks compared to F treatment (Figures 7, 8; Table 3).

**TABLE 3:** Key topological features of soil bacterial community co-occurrence networks under different treatments at different growth stages.

	Seedling stage				Maturity stage			
	BF	S	F	SF	BF	S	F	SF
Pos.edges (percentage)	62 (100)	100 (95)	76 (99)	110 (100)	76 (99)	49 (88)	40 (100)	62 (97)
neg.edges (percentage)	0 (0)	5 (5)	1 (1)	0 (0)	1 (1)	7 (12)	0 (0)	2 (3)
num.nodes	47	49	45	51	44	36	33	41
num.edges	62	105	77	110	77	56	40	64
num.vertices	47	49	45	51	44	36	33	41
average.degree	2.64	4.29	3.42	4.31	3.50	3.11	2.42	3.12
Connectance	0.06	0.09	0.08	0.09	0.08	0.09	0.08	0.08
average.path.length	2.42	3.77	4.77	3.05	2.64	3.74	3.59	2.67
graph.density	0.06	0.09	0.08	0.09	0.08	0.09	0.08	0.08
clustering.coefficient	0.57	0.47	0.55	0.51	0.53	0.36	0.59	0.63
Diameter	6	10	13	7	8	11	9	8
centralization.betweenness	0.05	0.19	0.26	0.10	0.05	0.24	0.13	0.04
centralization.degree	0.12	0.12	0.13	0.11	0.15	0.11	0.08	0.12

**FIGURE 9:**
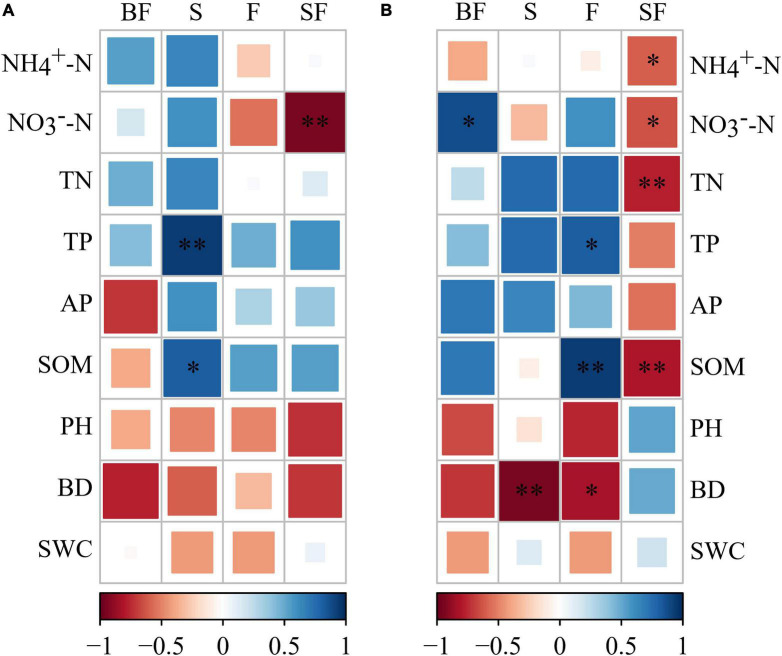
Correlation between keystone taxa and soil physicochemical properties under different treatments. **(A)** Seedling stage, **(B)** maturity stage. The darker the color, the stronger the correlation. BF, S, F and SF stand for: straw biochar with fertilizer, straw, fertilizer, and straw with fertilizer, respectively. NH_4_^+^-N, ammonium; NO_3_^–^-N, nitrate; TN, total nitrogen; TP, total phosphorus; AP, available phosphorus; SOM, soil organic matter; BD, bulk density; SWC, soil water content. *p < 0.05, ^**^p < 0.01.

The bacterial KT at SS and MS of maize were obtained in the four treatments (Table 4). We found that KT were significantly affected by growth stage in different treatments. Uncultured genus was the KT of the BF treatment and F treatment and was classified as Gemmatimonadetes. Solirubrobacter and Unclassified_nocardioidaceae were the KT in the S and SF treatments at SS, respectively. But they are both classified as Actinobacteria. Interestingly, we found that the KT in the BF, S, and F treatments all belonged to Actinobacteria at MS. The KT in the BF and F treatments were both Unclassified_micromonosporaceae. In addition, the KT in the SF treatment were Nitrospira and CCD24, which were quite different from the other three treatments. It indicated that biochar incorporation had little effect on bacterial KT, but straw incorporation significantly affected bacterial KT. We further analyzed the correlations between KT and environmental factors in the different treatments (Figure 9). At SS, the KT of S and SF treatments were significantly correlated with environmental factors (NO_3_^–^-N, TP, and SOM) (*p* < 0.05), while the other two treatments did not reach a significant level. The correlation of bacterial KT with environmental factors in the four treatments increased with maize growth. Specifically, KT in BF treatment were significantly correlated with NO_3_^–^-N (*p* < 0.05); KT in S treatment were significantly correlated with BD (*p* < 0.01); KT in F treatment were significantly correlated with SOM, TP and BD (*p* < 0.05); KT in SF treatment were significantly correlated with nitrogen indicators (NH_4_^+^-N, NO_3_^–^-N and TN) and SOM (*p* < 0.05).

**TABLE 4:** The keystone taxa in bacterial networks under different treatments at different growth stages.

Stage	Treatment	ASV_ID	Phylum	Genus	Degree	Closeness centrality	Betweenness centrality
Seedling stage	BF	ASV_114014	Gemmatimonadetes	Uncultured	8	0.030	61
	S	ASV_242313	Actinobacteria	Solirubrobacter	10	0.134	190
	F	ASV_114014	Gemmatimonadetes	Uncultured	9	0.088	26
	SF	ASV_66802	Actinobacteria	Unclassified_nocardioidaceae	10	0.067	50
Maturity stage	BF	ASV_228348	Actinobacteria	Unclassified_micromonosporaceae	10	0.044	37
	S	ASV_296676	Actinobacteria	Streptomyces	7	0.119	174
	F	ASV_113785	Actinobacteria	Unclassified_micromonosporaceae	5	0.059	78
	SF	ASV_92735	Nitrospirae	Nitrospira	8	0.040	26
		ASV_302974	Proteobacteria	CCD24	8	0.040	26

## Discussion

### Response of bacterial diversity to straw and straw biochar addition with maize growth

In this study, we found that the α-diversity and β-diversity of soil bacteria increased significantly from SS to MS, and the α-diversity of bacteria was significantly affected by treatment but not by soil depth. This suggests that certain bacterial groups have a greater ability to degrade stubborn recalcitrant compounds (Xiao et al., 2022). The soil depth and growth stage significantly affected bacterial β-diversity, according to ANOSIM analysis. It shows that changes in bacterial communities are seasonal and hierarchical. The same results were also obtained by Hellequin et al. (2021) and Yang et al. (2022). They concluded that the changes of the soil microbial community depended on the growth stage of plants and soil depth. The effect in the initial stage of plant growth is weak, and the greater the difference in soil depth, the stronger the effect. The relationship is greatest with the release of various root exudates in different growth stages of vegetation (Bello et al., 2021). Liu et al. (2022b) showed that temporal succession was the main driving force for community structure, and crop types and root exudates were the main reason for the differences in community composition during the succession period. On the other hand, the decomposition of crop SSB and the dissolution of chemical fertilizers lead to the release of many nutrients and soluble organic matter, which stimulate the growth of microorganisms and the development of diversity (Ge et al., 2021; Partovi et al., 2021). In addition, we found that bacterial diversity of the S treatment was slightly lower than that of the other treatments at SS, but higher than that of the other treatments at MS. Because the S treatment at SS did not provide sufficient nutrients for the growth of the crop, the growth rate was significantly reduced, resulting in a decrease in nutrients required by the crops, while the straw provided a carbon source for the microorganisms, increasing the advantage of competition for nutrients between microorganisms and plant for limited nutrients (Bei et al., 2018). The high nutrient requirement for maize growth in the F, SF, and BF treatments intensifies the nutrient competition between microorganisms and crops, and affects the growth of microorganisms, which may be negative (Bahram et al., 2020; Nevins et al., 2021). Meanwhile, it was also shown that the process of crops competing with microorganisms for limited nutrients is beneficial to crop growth in SF and BF treatments and enhances crop competitive advantage compared to F treatments, thus significantly reducing the bacterial diversity of SF and BF treatments. This is consistent with previous research findings (Lévesque et al., 2020). In summary, soil bacterial diversity is affected by the interaction of various factors such as crop growth status, growth stage, soil depth and treatment.

### Bacterial community structure and composition in response to straw and straw biochar with maize growth

Our study shows that the most abundant microbial phylum in soil is Actinobacteria, followed by Proteobacteria, Acidobacteria and Chloroflexi, which are considered to be the most common bacterial phyla in agricultural cultivation (Daquiado et al., 2016). But there are differences under different treatments, which is consistent with previous research results (Zhang and Lv, 2020). Actinobacteria and Acidobacteria are oligotrophic with lower growth rates, and prefer soil environments with low resource availability (Pascault et al., 2013; Liu et al., 2014). But the bacteria in Actinobacteria have the ability to produce lignocellulose, hydrolase and degrade lignocellulose, which play a particularly important role in degrading persistent compounds (Su et al., 2015; Bello et al., 2020). The relative abundance of Actinobacteria was reduced and the relative abundance of Proteobacteria and Acidobacteria increased with crop growth. Actinobacteria are a group of bacteria that easily grow on carbon-rich substances, and the decrease in Actinobacteria is attributed to the reduction of nutrients such as carbon sources along with the decomposition of straw and biochar, which limit the growth of bacteria (Ali et al., 2022). The relative abundance of Acidobacteria was decreased in SF and BF treatments. It is closely related to change in soil PH (Yin et al., 2021). This suggests that changes in bacterial survival patterns are based on resource and nutrient status acquired during decomposition (Carbonetto et al., 2014). The relative abundance of Proteobacteria increased in BF treatment compared with F and S treatments. Proteobacteria belong to eutrophic bacteria, and the decomposition of SSB provides a suitable environment for it to grow rapidly in the soil (Yang et al., 2022). In addition, the relative abundance of Chloroflexi was significantly increased in BF treatment and decreased in S and SF treatments. A possible explanation is that due to the small particle size of the straw biochar incorporated in this experiment, the soil bulk density slightly increased, forming an anaerobic environment, while the straw incorporation increased soil aeration, thereby reducing the relative abundance of Chloroflexi (Edeh and Mašek, 2021). We also found the effect of SSB addition on bacterial community composition at the bacterial genus. The species of bacterial genera and the number of unique genera were increased in BF and SF treatments compared to F treatment. The number of bacterial genera was increased in SF treatment compared with S treatment, indicating that fertilization can also alter bacterial community composition (Ge et al., 2021). Zhao et al. (2016) reported that the application of chemical fertilizers and straw returning to the field changed the structure of soil microbial community.

Previous studies have demonstrated that soil physicochemical properties are the main drivers affecting soil microbial composition and structure (Bai et al., 2020; Bello et al., 2021). It is well known that total organic carbon is the main energy source for microbial growth and activity, and significantly affects the composition and structure of microbial communities in soil (Xue et al., 2018; Araujo et al., 2021). Through RDA analysis, this study found that PH, SOM, and TN were the key factors that significantly affected bacterial community changes. The result corroborated the findings of Qi et al. (2021). Certain microorganisms can obtain C and N from SSB to grow and reproduce with appropriate C:N ratios (Li et al., 2017). Delgado-Baquerizo et al. (2018) concluded that soil PH was an important factor affecting regional soil bacterial communities. It also demonstrates that any significant deviation in pH can cause stress on unicellular microorganisms and also lead to changes in nutrients in the environment, which in turn control microbial composition and diversity in the ecosystem (Gui et al., 2022).

### Co-occurrence network and keystone taxa responses of soil bacterial communities

The bacterial networks of the S and SF treatments were similar, but very different compared to the BF and F treatments at SS (Figure 7; Table 3). The S and SF treatments increased the complexity of the network compared to the F treatment. With the growth of maize, the network complexity of BF treatment increases, while the other three treatments network complexity decreases. BF treatment had the most complex bacterial network at MS, followed by SF treatment (Figure 8; Table 3). The same result was obtained by Yu et al. (2018a). It has been reported that more complex networks may be formed in nutrient-rich environments (Yang et al., 2019). It also proved that the addition of SSB had a positive effect on soil properties in LCBS. Increasingly complex networks improve material cycling in soil and information exchange between bacteria, which means greater stability and resistance of the bacterial community (Karimi et al., 2017). Wagg et al. (2019) proposed that a more developed microbial co-occurrence network pattern implies increased soil ecosystem function related to carbon and nutrient cycling. Bacterial networks are dominated by positive interactions in the different treatments (Table 3), implying that bacteria are mutually beneficial or have similar functions (Yang et al., 2022). The number of positive correlation edges was significantly increased in SSB addition compared with F treatment, because the degradation of complex organic matter requires a variety of bacteria to work together (Deng et al., 2012; Yang et al., 2022). Recent studies have also shown that organically managed farmlands have more complex microbial networks and more keystone taxa (Banerjee et al., 2019).

The KT play specific roles in the cycling of different substances and have important effects on the structure and function of soil microorganisms (Banerjee et al., 2016; Yu et al., 2018a). KT were classified as Gemmatimonadetes and Actinobacteria at SS. KT were classified as Actinobacteria, Nitrospirae and Proteobacteria at MS, with Actinobacteria predominating. We believe that bacterial community succession may be affected by affecting KT at different growth stages of maize under SSB additions. This observation is consistent with existing studies (Sun et al., 2020). Gemmatimonadetes are thought to be more prone to oligotrophic taxa with higher C: N, which are related to the decomposition of complex organic matter, such as the initial stage of biochar returning to the field (Carbonetto et al., 2014). Actinobacteria are considered to mainly decompose straw and become a KT in straw treatment (Su et al., 2015). It is significantly correlated with organic matter and is extensively involved in carbon assimilation (Sun et al., 2020). Proteobacteria and Nitrospirae were significantly negatively correlated with N indices in the SF treatment. Zhang et al. (2021a) suggested that the addition of high nitrogen had a strong negative effect on Proteobacteria under straw returning. Nitrospirae, the most prevalent nitrite oxidizing bacteria, are primarily responsible for the biogeochemical nitrogen cycle and tend to thrive in low carbon and nitrogen conditions (Le Roux et al., 2016). But straw and fertilizer addition helped to increase soil TN and SOM content, which can stimulate decomposer activity, indicating that the coupled effect of carbon and nitrogen on Nitrospirae should be considered in future studies (Zhang et al., 2021b; Xiao et al., 2022). Notably, the same taxa were not observed to appear as KT in the bacterial networks of the different treatments. However, we found taxa from the same genus as the KT in multiple bacterial co-occurrence network patterns, namely ASV_114014, ASV_228348 and ASV_113785. These findings revealed that many species have similar functions (Han et al., 2022) and that KT are constantly changing with conditions (Bello et al., 2020; Ge et al., 2021). Interestingly, the bacterial KT Gemmatimonadetes and Nitrospirae had lower relative abundances. It shows that rare taxa are also very important in maintaining the microbial co-occurrence network. Similarly, previous studies have illustrated that rare species often have different functional characteristics than abundant species and make greater contributions to the ecosystem (Chapman et al., 2018; Kurm et al., 2019; Ma et al., 2021).

## Conclusion

This study determined the response of the bacterial community composition, structure and co-occurrence network pattern to the soil physicochemical properties under different combination applications in LCBS during maize growth. The dominant phyla of soil bacteria were Actinobacteria, Proteobacteria, Acidobacteria, and Chloroflexi in all treatments. Bacterial communities showed distinct seasonality and stratification, and the main drivers for this change from soil PH, SOM, and TN changes. Overall, SSB incorporation decreased bacterial community diversity under fertilization conditions. However, the incorporation of SSB stimulated positive information exchange and synergistic effects between soil bacteria and promoted soil material circulation. Thus, more complex and stable bacterial networks were induced, described by more nodes, edges and centrality. Not only that, the positive effect of straw biochar on microbial network patterns gradually increased with time. Finally, we identified bacterial keystone taxa with low relative abundance, which play an important role in maintaining soil ecological stability. The study highlights the importance of SSB addition to improve soil quality and maintain microbial diversity in LCBS.

## Data availability statement

The datasets presented in this study can be found in online repositories. The names of the repository/repositories and accession number(s) can be found below: NCBI, PRJNA861886.

## Author contributions

MD: data curation and writing—original draft. JZ: conceptualization, methodology, resources, and writing—review and editing. GW: conceptualization, writing—review and editing, and acquisition of funding. CL: formal analysis and supervision. ZW: data curation and supervision. All authors contributed to the article and approved the submitted version.
