# Supplementary material for: Response of bacterial community composition and co-occurrence network to straw and straw biochar incorporation
Source: Front Microbiol. 2022 Sep 30;13:999399. doi: 10.3389/fmicb.2022.999399 (PMC9563622; doi:10.3389/fmicb.2022.999399)
Supplement: Supplementary file 1 [file Data_Sheet_1.docx]

**Supplementary data**


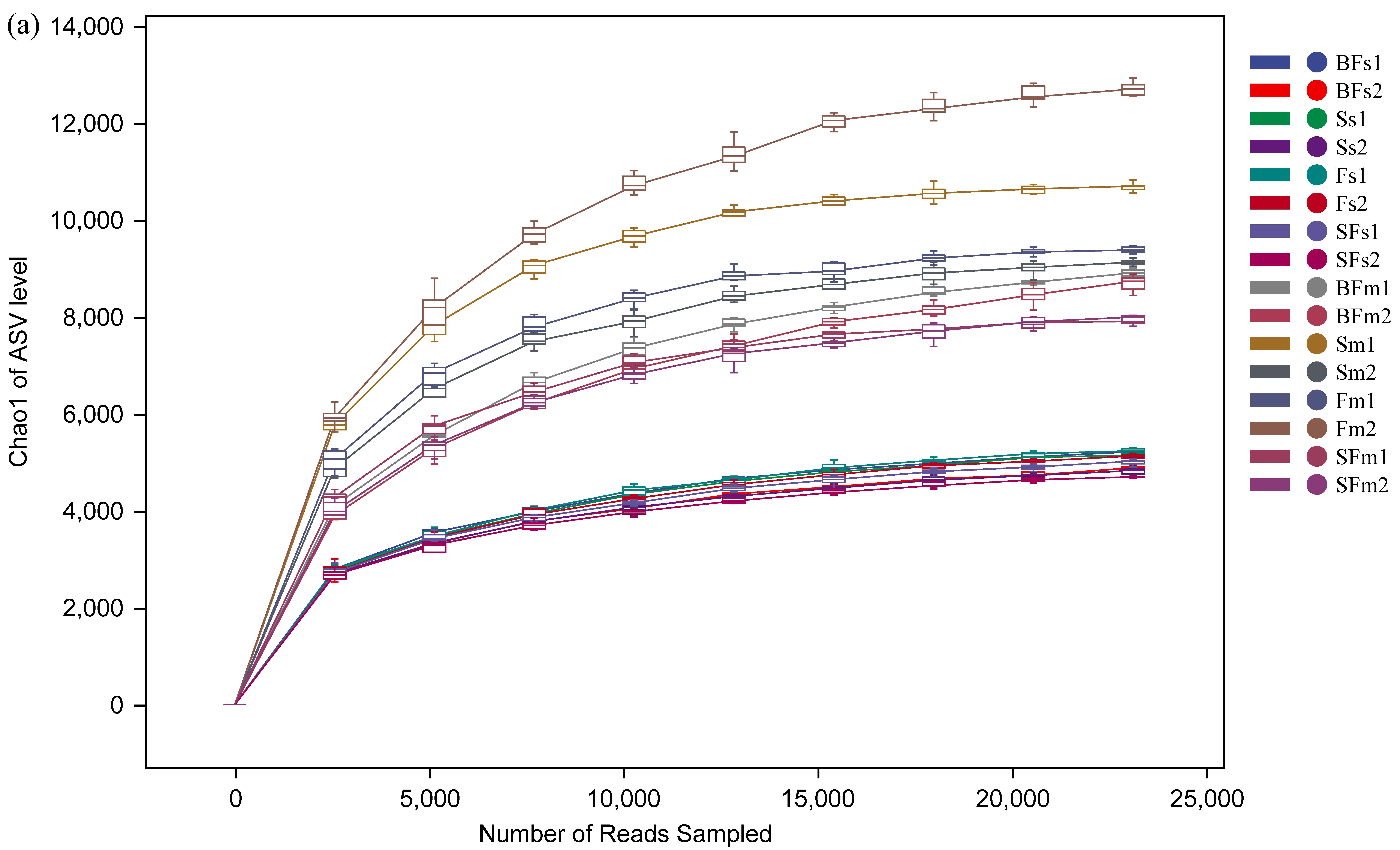


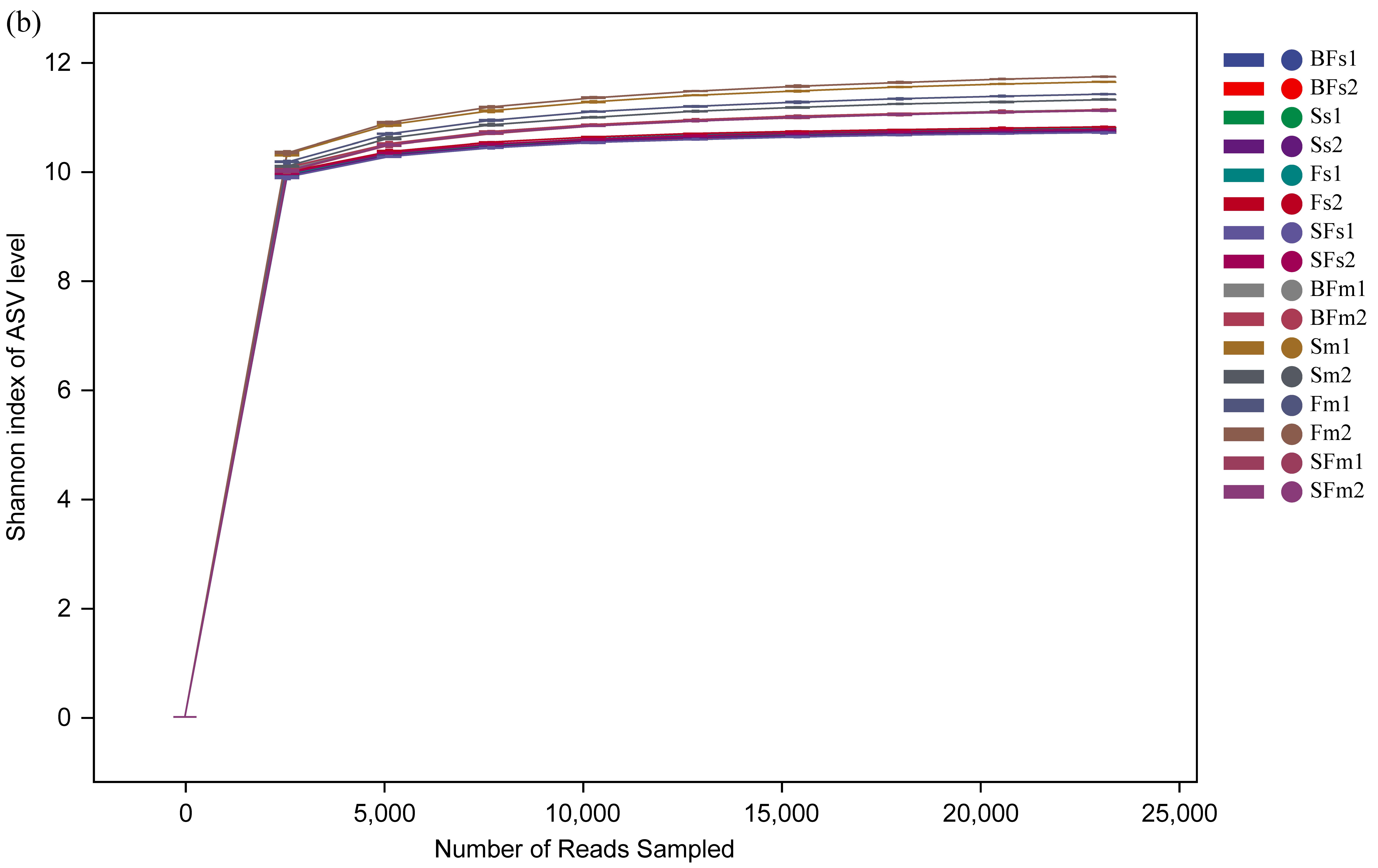


Figure S1 Rarefaction curves of soil bacterial communities. (a) Chao1, (b) Shannon index

Table S1 Significance test values for the effect of each environmental factor on the bacterial community at 0-10 cm depth

| Seedling stage | | | Maturity stage | | |
| --- | --- | --- | --- | --- | --- |
| Soil variable | r^2^ | p_values | Soil variable | r^2^ | p_values |
| NH_4_^+^-N | 0.198 | 0.392 | NH_4_^+^-N | 0.362 | 0.131 |
| TN | 0.453 | 0.064 | NO_3_^-^-N | 0.297 | 0.205 |
| TP | 0.022 | 0.907 | TN | 0.236 | 0.274 |
| AP | 0.43 | 0.084 | TP | 0.143 | 0.515 |
| SOM | 0.06 | 0.768 | AP | 0.009 | 0.964 |
| BD | 0.193 | 0.403 | SOM | 0.576 | 0.026 |
| SWC | 0.354 | 0.148 | PH | 0.422 | 0.074 |
|  |  |  | BD | 0.159 | 0.452 |
|  |  |  | SWC | 0.049 | 0.797 |

Table S2 Significance test values for the effect of each environmental factor on the bacterial community at 10-20 cm depth

| Seedling stage | | | Maturity stage | | |
| --- | --- | --- | --- | --- | --- |
| Soil variable | r^2^ | p_values | Soil variable | r^2^ | p_values |
| NH_4_^+^-N | 0.412 | 0.098 | NH_4_^+^-N | 0.032 | 0.833 |
| NO_3_^-^-N | 0.147 | 0.493 | NO_3_^-^-N | 0.342 | 0.184 |
| TN | 0.544 | 0.032 | TP | 0.286 | 0.187 |
| TP | 0.26 | 0.254 | AP | 0.338 | 0.168 |
| SOM | 0.361 | 0.14 | SOM | 0.022 | 0.913 |
| PH | 0.533 | 0.038 | PH | 0.285 | 0.206 |
| BD | 0.211 | 0.36 | BD | 0.039 | 0.802 |
|  |  |  | SWC | 0.211 | 0.38 |
